# A Risk Scoring Model for High-Dose Methotrexate-Induced Liver Injury in Children With Acute Lymphoblastic Leukemia Based on Gene Polymorphism Study

**DOI:** 10.3389/fphar.2021.726229

**Published:** 2021-09-29

**Authors:** Xia He, Pingli Yao, Mengting Li, Hong Liang, Yilong Liu, Shan Du, Min Zhang, Wenzhuo Sun, Zeyuan Wang, Xin Hao, Ze Yu, Fei Gao, Xinxia Liu, Rongsheng Tong

**Affiliations:** ^1^ Department of Pharmacy, Sichuan Academy of Medical Sciences and Sichuan Provincial People’s Hospital, Chengdu, China; ^2^ Personalized Drug Therapy Key Laboratory of Sichuan Province, School of Medicine, University of Electronic Science and Technology of China, Chengdu, China; ^3^ Ya’an Polytechnic College, Ya’an, China; ^4^ Hospital of Chengdu University of Traditional Chinese Medicine, Chengdu, China; ^5^ Xi’an Jiaotong-liverpool University, Xi’an, China; ^6^ Beijing Medicinovo Technology Co. Ltd., Beijing, China; ^7^ Dalian Medicinovo Technology Co. Ltd., Dalian, China

**Keywords:** acute lymphoblastic leukemia, high-dose methotrexate, liver injury, gene polymorphism, ridge regression model, children

## Abstract

A study on 70 acute lymphoblastic leukemia (ALL) children (age ≤16 years) treated with high-dose methotrexate (HD-MTX) in Sichuan Provincial People’s Hospital was conducted. The aim of the study was to establish a risk-scoring model to predict HD-MTX-induced liver injury, considering gene polymorphisms’ effects. Data screening was performed through *t*-test, chi-square test, and ridge regression, and six predictors were identified: age, *MTRR_AA, MTRR_AG, SLCO1B1_11045879_CC*, albumin_1 day before MTX administration, and IBIL_1 day before MTX administration (*p* < 0.1). Then, the risk-scoring model was established by ridge regression and evaluated the prediction performance. In a training cohort (*n* = 49), the area under the curve (AUC) was 0.76, and metrics including accuracy, precision, sensitivity, specificity, positive predictive value, and negative predictive value were promising (0.86, 0.81, 0.76, 0.91, 0.81, 0.88, respectively). In a test cohort (*n* = 21), the AUC was 0.62 and negative predictive value was 0.80; other evaluation metrics were not satisfactory, possibly due to the limited sample size. Ultimately, the risk scores were stratified into three groups based on their distributions: low- (≤48), medium- (49–89), and high-risk (>89) groups. This study could provide knowledge for the prediction of HD-MTX-induced liver injury and reference for the clinical medication.

## Introduction

Acute lymphoblastic leukemia (ALL) is a malignancy with high incidence in children aged between 1 and 5 years, which needs a long course of treatment (Preethi, 2014). In China, about 12,000 children aged below 16 are newly diagnosed with acute leukemia annually (Tang et al., 2008). Fortunately, due to the development of new drugs and precise chemotherapy, the outcome of pediatric ALL has been improved significantly over the past years; the 5-years survival rate is expected to increase up to 90% (Imanishi et al., 2007; Yang et al., 2012). High-dose methotrexate (HD-MTX) treatment during the consolidation phase is a major component in ALL treatment protocols (Erčulj et al., 2012). MTX is a folate reductase inhibitor and is stored in cells as polyglutamates (Fotoohi and Albertioni, 2008; Elbarbary et al., 2016). Being a result of long-term MTX treatment, the polyglutamates accumulate to higher levels, leading to a longer intracellular presence of the drug (Elbarbary et al., 2016). Previous reports demonstrate a variety of toxic reactions caused by HD-MTX, and liver injury is one of the serious adverse events (ADEs) (Schmiegelow, 2009; Conway and Carey, 2017). MTX-induced liver injury has been studied in patients aged >18 years with rheumatoid arthritis (RA); for instance, Japanese researchers investigated the risk factors for abnormal hepatic enzyme elevation by MTX in adult RA patients (Hakamata et al., 2018). However, the influencing factors of HD-MTX and its risk prediction model for ALL children have not been sufficiently explored.

**FIGURE 1:**
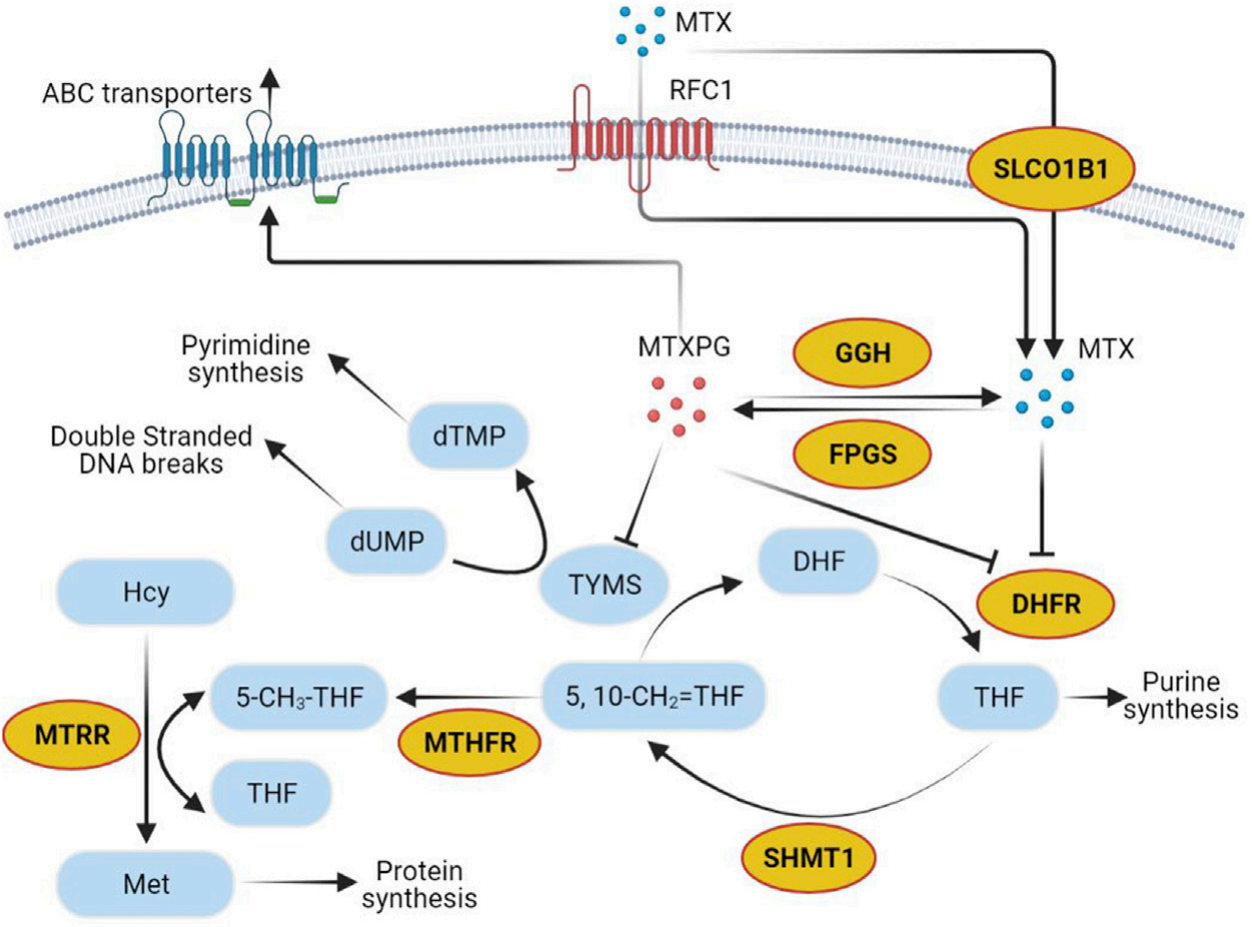
Methotrexate pathway. Yellow circles show genes selected in the present study. Abbreviations: MTX, methotrexate; RFC1, reduced folate carrier-1; SLCO1B1, solute carrier organic anion transporter family member 1B1; dTMP, deoxythymidine monophosphate; dUMP, deoxyuridine monophosphate; TYMS, thymidylate synthetase; MTXPG, the polyglutamated forms of MTX; GGH, gamma-glutamyl hydrolase; FPGS, folylpolyglutamate synthetase; Hcy, homocysteine; MTRR, methionine synthase reductase; Met, Methionine; 5-CH3-THF, 5-methyltetrahydrofolate; THF, tetrahydrofolate; MTHFR, methylenetetrahydrofolate reductase; 5,10-CH2 = THF, 5,10-methylenetetrahydrofolate; DHF, dihydrofolate; DHFR, dihydrofolate reductase; SHMT1, serine hydroxymethyl transferase 1.

Most ADEs of MTX treatment show individual differences, which can be partly explained by the gene sequence variation of proteins or transporters during the metabolism or excretion of MTX (Schmiegelow, 2009; Mikkelsen et al., 2011; Csordas et al., 2014; Moriyama et al., 2015). Recently, polymorphisms in genes are believed important to MTX pharmacokinetics, affecting MTX toxicity by altering the expression and activities of folate pathway enzymes (Csordas et al., 2014). Several genes play key roles in the MTX metabolism and transport pathway (Figure 1). MTX can be transformed into MTX polyglutamic (MTXPGs) with higher activity and toxicity, which is mediated by folylpolyglutamate synthetase (FPGS) and gamma-glutamyl hydrolase (GGH) (Fotoohi and Albertioni, 2008). Both MTX and MTXPGs combine with dihydrofolate reductase (DHFR) that blocks the reduction of dihydrofolate to tetrahydrofolate competitively (Fotoohi and Albertioni, 2008). As an intermediate metabolite, tetrahydrofolate can be changed into methylenetetrahydrofolate and methyltetrahydrofolate via serine hydroxymethyl transferase 1 (SHMT1) and methylenetetrahydrofolate reductase (MTHFR), correlating to MTX hepatic toxicity (Schmiegelow, 2009). There are also reports indicating that certain polymorphisms in methionine synthase reductase (MTRR) can decrease homocysteine levels and increase folate and cobalamin levels (Lv et al., 2018). Additionally, solute carrier organic anion transporter 1B1 (SLCO1B1) has been described as a membrane transporter involved in the clearance of MTX (Lopez-Lopez et al., 2011; Niemi et al., 2011). Clarifying the relationship between gene polymorphisms and HD-MTX-induced liver injury will be helpful for risk prediction and individualized optimal therapy (Kantar et al., 2009).

## Methods

### Study Population

A total of 70 hospitalized Chinese Han children aged 1–16 years were enrolled in this study and were diagnosed with ALL and treated in Sichuan Provincial People’s Hospital from October 2015 to August 2018. The specific diagnostic criteria and ALL risk classification were administered according to the Recommendations for Diagnosis and Treatment of Childhood Acute Lymphoblastic Leukemia (Version 4.0). Pediatric ALL patients received regimens referring to the widely used Chinese Children’s Leukemia Group (CCLG)-ALL 2008 protocols (Cui et al., 2018).

**FIGURE 2:**
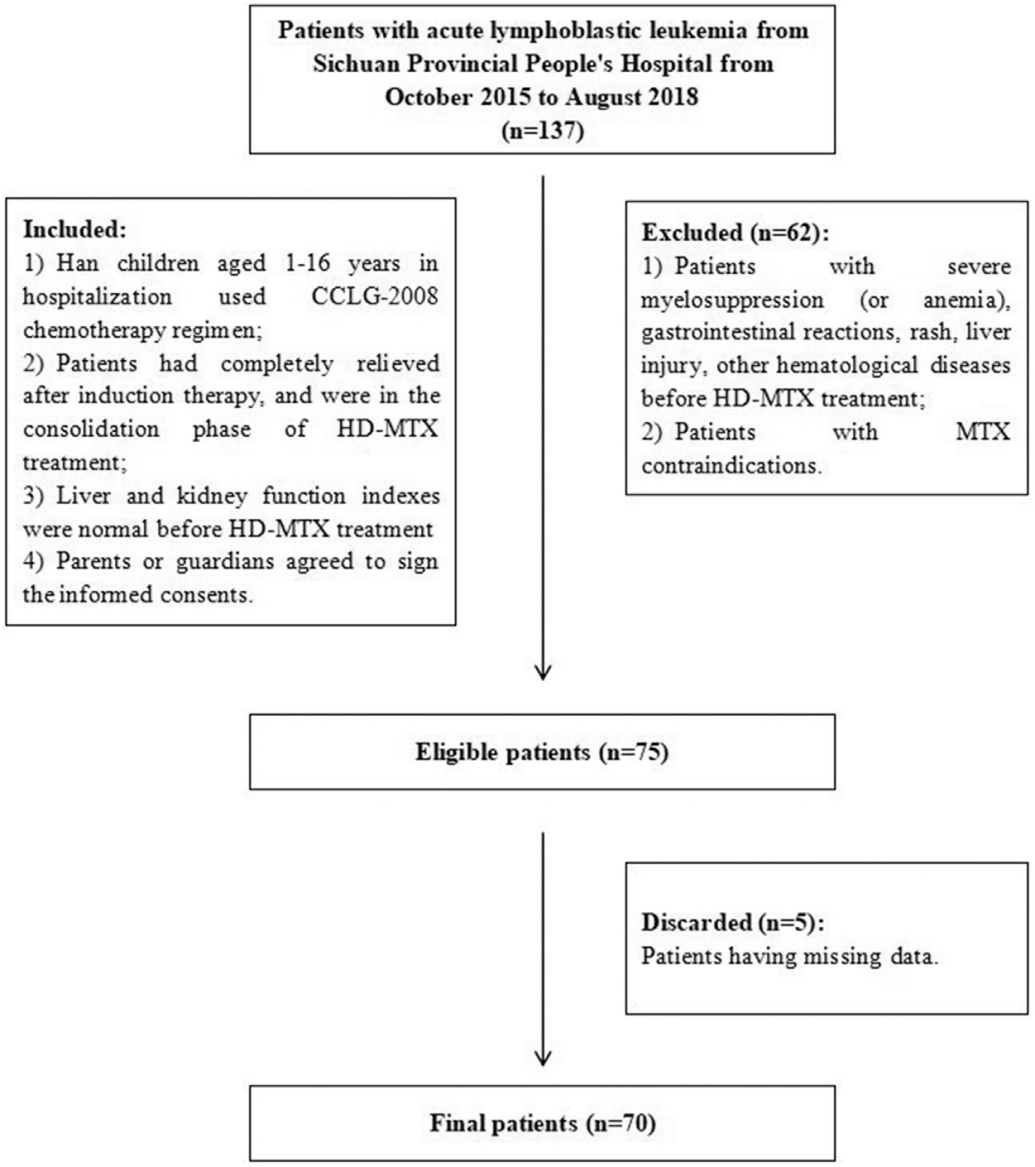
Flowchart of patient inclusion. Abbreviations: CCLG-2008, Chinese Children’s Leukemia Group-2008 protocols; MTX, methotrexate; HD-MTX, high-dose methotrexate.

The inclusion criteria were 1) Han children aged 1–16 years who were hospitalized and used the CCLG-2008 chemotherapy regimen; 2) all patients were completely relieved after induction therapy and were in the consolidation phase of HD-MTX treatment; 3) their liver and kidney function indexes were normal before HD-MTX treatment; 4) parents or guardians agreed to sign the informed consent. Among all candidates, those with severe myelosuppression (or anemia), gastrointestinal reactions, rash, liver injury, other hematological diseases before HD-MTX treatment and MTX contraindications were excluded. The workflow of selecting eligible patients is displayed in Figure 2. The study population was randomly divided into training and test cohorts (7:3). All patients were followed up for clinical and laboratory parameters because they received HD-MTX treatment for the first time to assess the development of liver injury. All demographic, clinical, laboratory, and medication data were obtained as the input variables.

This study was approved by the Ethics Committee of Sichuan Provincial People’s Hospital. In all cases, informed consent was obtained from the parents or guardians of each participant in advance. Our study is registered in the Clinical Trial Management Public Platform (ChiCTR1800015307).

### MTX Treatment and Toxicity Assessment

According to the Recommendations for Diagnosis and Treatment of Childhood Acute Lymphoblastic Leukemia (Version 4.0), MTX for injection (Jiangsu Hengrui Pharmaceutical Co., LTD., National Drug Approval Number H32026197, 1g/dose) was given at 5.0 g/m^2^ to the patients in the medium- and high-risk grades, dosage of MTX was 2.0 g/m^2^ in the low-risk group, and both were calculated by body surface area. One tenth of the total MTX dose was administered as an assault dose in the first 30 min at a rapid intravenous drip, and the remaining dose was administered at a constant intravenous drip in the following 23.5 h. After HD-MTX administration, leucovorin (Jiangsu Hengrui Pharmaceutical Co., LTD., National Drug Approval Number H32022391, 100 mg/dose) was administered at 15 mg/m^2^ per time as rescue therapy to reduce the toxicity at 42, 48, and 54 h, respectively, until MTX blood concentration was ≤0.1 mol/L. If patients had delayed elimination, leucovorin at the same dose was given 1–3 times (Q6h) as supplements. Hydration and alkalinization were required 3 days before and after HD-MTX treatment. The venous blood was collected in the anticoagulant tube at 48 and 72 h after intravenous infusion of MTX. The plasma concentration of MTX was determined by homogeneous enzyme amplification immunoassay (Viva-E, Siemens).

The identification of liver injury used alanine transaminase (ALT) and/or aspartate aminotransferase (AST) as indexes. The normal value of both ALT and AST is 0–40 U/L. According to the National Cancer Institute Common Terminology Criteria for Adverse Events (NCI-CTCAE v4.03) scale, hepatic toxicity of severity grade 2 and above (grade 2, medium; grade 3, severe; grade 4, life-threatening; and grade 5, fatal) is considered as liver injury (National Institute of Can, 2009). To be specific, hepatotoxicity over grade 2 is defined as the levels of ALT and/or AST over the upper limit of the normal value (40 U/L) by three to five times with no symptoms or exceeding the upper limit of the normal value by three times with aggravated symptoms, such as fatigue, nausea, vomiting, pain or tenderness in the upper right abdomen, fever, rash, and eosinophilia (National Institute of Can, 2009). ALT and AST were measured 1 day before and 3 and 7 days after HD-MTX administration (Beckman AU5800).

### Variable Selection

#### Genotyping Analysis

Because of the prominent contribution to MTX toxicity among individuals, we analyzed up to 10 polymorphisms in seven genes (*MTHFR, MTRR, SLCO1B1, FPGS, GGH, SHMT1,* and *DHFR*) from the MTX metabolism and transport pathway.

In this study, real-time fluorescence quantitative polymerase chain reaction (PCR) was used to detect the gene analysis of patients. First, the blood genome column small volume extraction kit (Beijing Kangwei Century Biotechnology Co., LTD.) was used to extract the peripheral blood DNA of the children in strict accordance with its instructions. The concentration and purity of DNA were detected by NanoDrop 2000 (Thermo Fisher Scientific, United States) [DNA concentration was 10–30 ng/μl; purity (*A*_260 nm_/*A*_280 nm_) was 1.6–2.0]. The PCR instrument was ABI 7500 real-time quantitative PCR (Thermo Fisher Scientific, United States). MTHFR rs1801133, 1801131, MTRR rs1801394, SLCO1B1 rs2806283, and rs4149056 used gene test kits as follows: *MTHFR (C677T)* gene test kit (National instrument Approval Word 20173401322), *MTRR* and *MTHFR (A1298C)* gene test kit (Hubei Food and Drug Supervision Equipment Production License 20120580), *SLCO1B1* and *ApoE* gene test kit (National Instrument Approval License 20153400245; Wuhan Youzhiyou Medical Technology Co., LTD.). Primers and probes were designed and synthesized by Wuhan Youzhiyou Medical Technology Co., LTD., which is illustrated in Supplementary Table S1. PCR reaction system (25 μl): DNA template 1 μl and amplification reagent 24 μl (including PCR buffer, dNTPs, specific primer and probe, internal primer and probe, Taq enzyme, UNG enzyme); reaction conditions: pretreatment at 37°C for 10 min, predenaturation at 95°C for 5 min, denaturation at 95°C for 15 s, annealing at 60°C for 60 s (*MTHFR* and *MTRR*)/45s (*SLCO1B1*), a total of 40 cycles. We purchased TaqMan™ single nucleotide polymorphism (SNP) Genetyping Assays to detect loci: *SLCO1B1 rs11045879* (ID: C_31106904_10), *FPGS rs11545078* (ID: C_25623170_10), *GGHS rs11545078* (ID: C_25623170_10), *SHMT1 rs1979277* (ID: C_3063127_10), *DHFR rs408626* (ID: C_921,481_20). Reaction system (total 25 μl): DNA template 1 μl, Master Mix 12.5 μl, 20×TaqMan SNP Genotyping Assay working fluid 1.25 μl, supplemented with ddH2O 25 μl; reaction conditions: pretreatment at 60°C for 20 s, predenaturation at 95°C for 5 min, denaturation at 95°C for 5 s, annealing at 60°C for 60 s, a total of 40 cycles; extended at 60°C for 5 min.

#### Nongenetic Analysis

There are some nongenetic factors relating to hepatotoxicity. In addition to demographic factors (age, gender, height, weight, BMI, and body surface), some laboratory indexes were included for screening, such as complete blood counts, hemoglobin, albumin, bilirubin, ALT, AST, alkaline phosphatase (ALP) and gamma glutamyl transferase (GGT). The indicators for renal function were taken into consideration as well, including creatinine (Cr), blood urea nitrogen (BUN), urine protein, and urine pH. MTX dosage and plasma concentration at 48 and 72 h were also influencing factors of MTX toxicity.

### Model Construction and Evaluation

Ridge regression was applied to construct the model, which is a parameter estimation method and can address the collinearity problem for multiple linear regressions without reducing variables from the original data set (Mcdonald, 2010). On the other side, to achieve better estimation of the model coefficients, ridge regression can eliminate the bias of the correlations between variables. The regression coefficient *β*_*i*_ of each variable in ridge regression was weighted through the following formula:

$$\overset{\wedge }{\beta_{i}}=round\left(\frac{\beta_{i}}{\min\left(\left|\beta_{1,...,K}\right|\right)}/2\right)$$

After weighting the regression coefficient for each predictor, we got the risk-score calculation formula for liver injury. The cumulative risk score for each patient was calculated from the formula by summation of these weightings for respective predictors. Higher score indicates greater risk of liver injury. Risk groups for liver injury were stratified according to the distribution of patients’ total risk scores. On this basis, we set the score less than the lower quartile as low-risk (LR) level, score between the lower and upper quartile as medium-risk (MR) level, and score higher than the upper quartile as high-risk (HR) level. Model performance was evaluated through the receiver operating characteristic (ROC) curve and the value of area under the curve (AUC), which represent the overall ability of classification and prediction. Additional statistics, such as accuracy, sensitivity, specificity, positive predictive value, and negative predictive value were obtained.

### Statistical Analysis

Based on the significant variables after preliminary screening, the categorical variables were binarized with one-hot encoding. Univariate analysis was performed through two independent sample *t*-tests on continuous variables and a chi-square test on categorical variables with significance level at *p* < 0.1. After that, the important variables were further selected by ridge regression with a ranking of importance scores. Subsequently, the random forest (RF) method was used to fill in the missing values. RF shows the ability of imputing missing data into the given data set with high accuracy and less computation time (Pantanowitz and Marwala, 2009). Then, *t*-tests and chi-square tests were applied to verify the differences between variable characteristics of the training and test cohorts. Ridge regression was used to establish the risk scoring model. Ultimately, the distribution of three risk groups was given, and intergroup differences were assessed by chi-square test.

Data were collected, coded, and entered to the Statistical Package for the Social Sciences software version 22.0 and Python 3.7.0.

## Results

### Baseline Information

A total of 70 children with ALL were enrolled in our study, including 45 males (64.3%) and 25 females (35.7%), and the median age was 7.5 in a range between 1 and 16 years. The ratio of patients with to those without liver injury was about 1:2. In total, we collected 45 variables from clinical data and genotypes; the basic characteristics are listed in Tables 1, 2. The majority of patients had B-cell ALL (64.3%), and 67 patients were classified as MR or HR grade (95.7%). The median MTX dose administered for patients was 4 [interquartile range (IQR) 2.5–5.0] g, the median MTX concentration measured at 48 h was 0.39 (IQR 0.25–0.64) umol/l, and 33 patients (47.1%) had MTX concentration measured at 72 h > 0.1 umol/l. The detailed MTX plasma concentration values are shown in Supplementary Table S2. The median ALT and AST tested 1 day before HD-MTX treatment were 22.0 (IQR 14.0–36.8) U/L and 32.0 (IQR 23.0–38.0) U/L, respectively. After starting the MTX therapy regimen, liver injury occurred in 32.9% (*n* = 23) of the patients. The basic genotype information and distribution are shown in Table 2. According to the calculated results, the polymorphisms included in the model were in Hardy–Weinberg equilibrium, which means samples in our study are representative.

**TABLE 1:** Baseline characteristics of study population.

Variable	Value
Target variable	
Liver injury, n (%)	23 (32.9%)
Demographic information	
Age, year, median (IQR)	7.5 (4–12)
Gender, n (%)	
Male	45 (64.3%)
Female	25 (35.7%)
Height, cm, median (IQR)	119.5 (99.3–154.5)
Weight, kg, median (IQR)	24.8 (15.0–40.8)
Body surface area, m^2^, median (IQR)	0.9 (0.6–1.3)
BMI, kg/m^2^, median (IQR)	16.8 (15.7–18.8)
MTX information	
Dose, g, median (IQR)	4.0 (2.5–5.0)
C_48h_, umol/l, median (IQR)	0.39 (0.25–0.64)
C_48h_ after dose correction, umol/l, median (IQR)	0.13 (0.08–0.28)
C_72h_ ≤ 0.1 μmol/L, n%	37 (52.9%)
C_72h_ > 0.1 μmol/L, n%	33 (47.1%)
ALL information	
Immunophenotype, n (%)	
B-cell	45 (64.3%)
T-cell	12 (17.1%)
Others	13 (18.6%)
Risk grade	
LR	3 (4.3%)
MR	18 (25.7%)
HR	49 (70.0%)
Assay index	
WBC count_1 day before MTX administration, 10^9^/L, median (IQR)	3.3 (2.3–5.5)
NEU count_1 day before MTX administration, 10^9^/L, median (IQR)	1.5 (0.8–2.7)
LYM count_1 day before MTX administration, 10^9^/L, median (IQR)	1.2 (0.8–1.6)
EOS count_1 day before MTX administration, 10^9^/L, median (IQR)	0.019 (0.000–0.078)
BASO count_1 day before MTX administration, 10^9^/L, median (IQR)	0.010 (0.000–0.030)
RBC count_1 day before MTX administration, 10^12^/L, median (IQR)	3.2 (2.7–3.7)
PLT count_1 day before MTX administration, 10^9^/L, median (IQR)	239.5 (176.3–408.0)
Hb_1 day before MTX administration, g/L, median (IQR)	98.0 (83.3–107.0)
ALT_1 day before MTX administration, U/L, median (IQR)	22.0 (14.0–36.8)
AST_1 day before MTX administration, U/L, median (IQR)	32.0 (23.0–38.0)
Cr_1 day before MTX administration, μmol/L, median (IQR)	26.2 (20.8–37.5)
BUN_1 day before MTX administration, mmol/L, median (IQR)	3.8 (2.7–4.6)
Albumin_1 day before MTX administration, g/L, median (IQR)	41.9 (38.7–44.9)
Globin_1 day before MTX administration, g/L, median (IQR)	20.2 (17.9–23.8)
TP_1 day before MTX administration, g/L, median (IQR)	62.6 (59.0–57.5)
Globin/Albumin_1 day before MTX administration, median (IQR)	2.1 (1.7–2.4)
ALP_1 day before MTX administration, U/L, median (IQR)	155.0 (115.0–212.0)
GGT_1 day before MTX administration, U/L, median (IQR)	21.0 (13.0–40.0)
Urine protein_1 day before MTX administration, g, median (IQR)	0 (0–0)
Urine pH_1 day before MTX administration, median (IQR)	7.5 (7.5–8.0)
TBIL_1 day before MTX administration, μmoI/L, median (IQR)	9.6 (6.7–15.8)
DBIL_1 day before MTX administration, μmoI/L, median (IQR)	3.1 (2.3–4.3)
IBIL_1 day before MTX administration, μmoI/L, median (IQR)	7.0 (4.3–10.8)

**Abbreviation**: BMI, body mass index; MTX, methotrexate; ALL, acute lymphoblastic leukemia; C48h, 48-h blood concentration; C72h, 72-h blood concentration; LR, low-risk; MR, medium-risk; HR, high-risk; WBC, white blood cells; NEU, neutrophil; LYM, lymphocyte; EOS, eosnophils; BASO, basophils; RBC, red blood cells; PLT, platelet; Hb, hemoglobin; ALT, alanine transaminase; AST, aspartate aminotransferase; Cr, creatinine; BUN, blood urea nitrogen; A/G, the ratio of albumin to globin; TP, total protein; ALP, alkaline phosphatase; GGT, gamma glutamyl transpeptidase; TBIL, total bilirubin; DBIL, direct bilirubin; IBIL, indirect bilirubin.

**TABLE 2:** Basic information and distribution of genotypes.

Genes (RS no.)	Genotype	N (%)	Allele	Frequency	Hardy–Weinberg equilibrium	
					X^2^	*p*
MTHFR (rs1801133)	CC	25 (35.7%)	C	0.62	1.07	0.59
	CT	37 (52.9%)	C^a^	0.71		
	TT	8 (11.4%)	T	0.38		
			T^a^	0.29		
MTHFR (rs1801131)	AA	38 (54.3%)	A	0.74	0.15	0.93
	AC	28 (40.0%)	A^a^	0.84		
	CC	4 (5.7%)	C	0.26		
			C^a^	0.16		
SLCO1B1 (rs11045879)	CC	10 (14.3%)	C	0.41	0.63	0.73
	CT	37 (52.9%)	C^a^	0.45		
	TT	23 (32.9%)	T	0.59		
			T^a^	0.55		
SLCO1B1 (rs2306283)	AA	2 (2.9%)	A	0.20	0.36	0.84
	AG	24 (34.3%)	A^a^	0.21		
	GG	44 (62.9%)	G	0.80		
			G^a^	0.80		
SLCO1B1 (rs4149056)	TT	54 (77.1%)	T	0.86	6.3	0.04
	CT	12 (17.1%)	T^a^	0.88		
	CC	4 (5.7%)	C	0.14		
			C^a^	0.12		
FPGS (rs1544105)	CC	10 (14.3%)	C	0.32	2.3	0.32
	CT	25 (35.7%)	C^a^	0.31		
	TT	35 (50.0%)	T	0.68		
			T^a^	0.70		
GGH (rs11545078)	GG	65 (92.9%)	G	0.96	0.1	0.95
	AG	5 (7.1%)	G^a^	0.92		
	AA	0 (0.0%)	A	0.04		
			A^a^	0.08		
SHMT1 (rs1979277)	GG	59 (84.3%)	G	0.92	0.51	0.78
	AG	11 (15.7%)	G^a^	0.95		
	AA	0 (0.0%)	A	0.08		
			A^a^	0.05		
DHFR (rs408626)	TT	4 (5.7%)	T	0.32	3.14	0.21
	CT	37 (52.9%)	T^a^	0.35		
	CC	29 (41.4%)	C	0.68		
			C^a^	0.65		
MTRR (rs1801394)	AA	31 (44.3%)	A	0.67	0.09	0.96
	AG	32 (45.7%)	A^a^	0.73		
	GG	7 (10.0%)	G	0.33		
			G^a^	0.27		

a Reference allele, data from the PharmGKB Drug Genome Library of the East Asian, a sample of over 3,000 people, https:/www.pharmgkb.org.

b *p* > .05 showed data was accord with the Hardy–Weinberg equilibrium.

Abbreviations: RS, reference single nucleotide polymorphism.

### Selected Variables

The outcome of univariate analysis through *t*-tests and chi-square tests is illustrated in Table 3, which demonstrates the relationships of all variables to the liver injury. After data screening, seven variables were identified to make statistically significant contributions to liver injury (*p* < 0.1), including age, *MTRR_AA, MTRR_AG, SLCO1B1_11045879_CC*, lymphocyte (LYM) count_1 day before MTX administration, albumin_1 day before MTX administration, and indirect bilirubin (IBIL)_1 day before MTX administration with *p* values of 0.091, 0.003, 0.001, 0.096, 0.019, 0.094, and 0.068, respectively (shown in Table 3). Subsequently, importance scores of the seven variables were calculated and ranked through ridge regression, which were *MTRR_AG* (1.349)*, SLCO1B1_11045879_CC* (0.963)*,* albumin_1 day before MTX administration (0.730), *MTRR_AA* (0.558), age (0.443), IBIL_1 day before MTX administration (0.348), and LYM count_1 day before MTX administration (0.032) in descending order. Because LYM count_1 day before MTX administration had the lowest importance score that differed greatly from other variables and for the purpose of model simplicity, it was excluded as the final predictor. The top six variables were applied as predictors for liver injury, including age, *MTRR_AA, MTRR_AG, SLCO1B1_11045879_CC*, albumin_1 day before MTX administration, and IBIL_1 day before MTX administration. After interpolation of missing values via RF, a comparison of important variable characteristics between training and test cohorts was established (Table 4). At the confidence level of 0.05, there was no significant difference between the characteristics of each variable in the training and test cohorts.

**TABLE 3:** Significance analysis of the influencing variables of liver injury.

Variable	*t* statistics	χ^2^ statistics	*p*-value	Odds ratio (95% CI)
Age	−1.71	—	0.091	
Gender	—	0.17	0.676	1.246 (0.444–3.496)
Height	−0.43	—	0.671	
Weight	−0.52	—	0.606	
Body surface area	−0.48	—	0.630	
BMI	−0.80	—	0.426	
Dosage	−1.45	—	0.150	
C_48h_	−0.187	—	0.852	
C_48h_ after dose correction	0.471	—	0.639	
C_72h_	—	0.88	0.348	0.616 (0.223–1.698)
Immunophenotype	—			
B-cell	—	0.01	0.909	1.063 (0.374–3.019)
T-cell	—	0.001	0.969	1.026 (0.274–3.840)
Others	—	0.03	0.859	0.889 (0.242–3.262)
Risk grade	—			
LR	—	1.53	0.216	
MR	—	0.28	0.594	0.726 (0.223–2.362)
HR	—	1.11	0.291	1.852 (0.582–5.927)
*MTHFR_C677T*	—			
*CC*	—	0.01	0.909	0.941 (0.331–2.674)
*CT*	—	0.19	0.667	1.246 (0.457–3.398)
*TT*	—	0.25	0.615	0.651 (0.121–3.508)
*MTHFRA1298C*	—			
*AA*	—	0.06	0.804	0.881 (0.324–2.395)
*AC*	—	0.17	0.678	1.239 (0.450–3.412)
*CC*	—	0.12	0.730	0.667 (0.065–6.786)
*MTRR*	—			
*AA*	—	8.87	0.003	4.876 (1.657–14.350)
*AG*	—	11.07	0.001	0.143 (0.042–0.487)
*GG*	—	0.35	0.553	1.613 (0.329–7.893)
*SLCO1B1_11045879*	—			
*CC*	—	2.76	0.096	0.192 (0.023–1.618)
*CT*	—	2.10	0.147	2.131 (0.759–5.979)
*TT*	—	0.09	0.763	0.848 (0.290–2.480)
SLCO1B1*1b (rs2306283_A > G)	—			
*AA*	—	1.01	0.316	0.957 (0.901–1.017)
*AG*	—	0.004	0.951	1.033 (0.362–2.950)
*GG*	—	0.08	0.775	1.164 (0.411–3.294)
SLCO1B1*5 (rs4149056_T > C)	—			
*TT*	—	0.58	0.446	1.629 (0.461–5.752)
*TC*	—	0.001	0.969	1.026 (0.274–3.840)
*CC*	—	2.08	0.150	0.915 (0.838–0.998)
*FPGS (rs1544105_C > T)*	—			
*CC*	—	0.04	0.835	0.857 (0.200–3.673)
*CT*	—	0.17	0.676	1.246 (0.444–3.496)
*TT*	—	0.07	0.799	0.878 (0.324–2.384)
*GGH (rs11545078_A > G)*	—	0.40	0.525	0.489 (0.051–4.639)
*SHMT1 (rs1979277_A > G)*	—	1.27	0.259	0.402 (0.079–2.036)
*DHFR (rs408626_T > C)*	—			
*TT*	—	0.12	0.730	0.667 (0.065–6.786)
*TC*	—	1.21	0.271	0.570 (0.208–1.560)
*CC*	—	1.63	0.202	1.925 (0.700–5.294)
WBC count_1 day before MTX administration	−0.2	—	0.842	
NEU count_1 day before MTX administration	0.07	—	0.949	
LYM count_1 day before MTX administration	0.10	—	0.019	
EOS count_1 day before MTX administration	1.07	—	0.289	
BASO count_1 day before MTX administration	−1.16	—	0.251	
RBC count_1 day before MTX administration	−0.93	—	0.363	
PLT count_1 day before MTX administration	0.06	—	0.954	
Hb_1 day before MTX administration	−0.01	—	0.995	
ALT_1 day before MTX administration	−0.69	—	0.494	
AST_1 day before MTX administration	−0.91	—	0.364	
Cr_1 day before MTX administration	0.88	—	0.385	
BUN_1 day before MTX administration	0.73	—	0.465	
Albumin_1 day before MTX administration	1.70	—	0.094	
Globin_1 day before MTX administration	−0.06	—	0.953	
TP_1 day before MTX administration	1.28	—	0.206	
A/G_1 day before MTX administration	0.64	—	0.526	
ALP_1 day before MTX administration	0.24	—	0.808	
GGT_1 day before MTX administration	−0.81	—	0.429	
Urine protein_1 day before MTX administration	−0.56	—	0.584	
Urine pH_1 day before MTX administration	−0.07	—	0.942	
TBIL_1 day before MTX administration	−1.58	—	0.128	
DBIL_1 day before MTX administration	−1.45	—	0.164	
IBIL_1 day before MTX administration	−1.86	—	0.068	

**Abbreviations**: CI, confidence interval; BMI, body mass index; MTX, methotrexate; ALL, acute lymphoblastic leukemia; C48h, 48-h blood concentration; C72h, 72-h blood concentration; LR, low-risk; MR, medium-risk; HR, high-risk; MTHFR, methylenetetrahydrofolate reductase; SLCO1B1, solute carrier organic anion transporter 1B1; FPGS, folypolyglutamate synthetase; GGH, gamma-glutamyl hydrolase; DHFR, dihydrofolate reductase; SHMT1, serine hydroxymethyl transferase 1; MTRR, methionine synthase reductase; WBC, white blood cells; NEU, neutrophil; LYM, lymphocyte; EOS, eosinophils; BASO, basophils; RBC, red blood cells; PLT, platelet; Hb, hemoglobin; ALT, alanine transaminase; AST, aspartate aminotransferase; Cr, creatinine; BUN, blood urea nitrogen; A/G, the ratio of albumin to globin; TP, total protein; ALP, alkaline phosphatase; GGT, gamma glutamyl transpeptidase; TBIL, total bilirubin; DBIL, direct bilirubin; IBIL, indirect bilirubin.

**TABLE 4:** Comparison of significant variable characteristics between training and test cohorts.

Variable	Training cohort (n = 49)	Test cohort (n = 21)	*p*-value
Liver injury, n%			0.617
0	32 (65.3%)	15 (71.4%)	
	17 (34.7%)	6 (28.6%)	
Age, median (IQR)	8 (4–12)	7 (4–11)	0.191
*MTRR_AA*, n%			0.875
0	27 (55.1%)	12 (57.1%)	
1	22 (44.9%)	9 (42.9%)	
*MTRR_AG*, n%			0.173
0	24 (49.0%)	14 (66.7%)	
1	25 (51.0%)	7 (33.3%)	
*SLCO1B1_11045879_CC*, n%			0.456
0	43 (87.8%)	17 (81.0%)	
1	6 (12.2%)	4 (19.0%)	
Albumin_1 day before MTX administration, median (IQR)	41.9 (39.0–44.5)	42.7 (40.1–44.2)	0.102
IBIL_1 day before MTX administration, median (IQR)	7.2 (4.4–10.7)	5.4 (4.3–8.0)	0.096

### Model Construction and Evaluation

**FIGURE 3:**
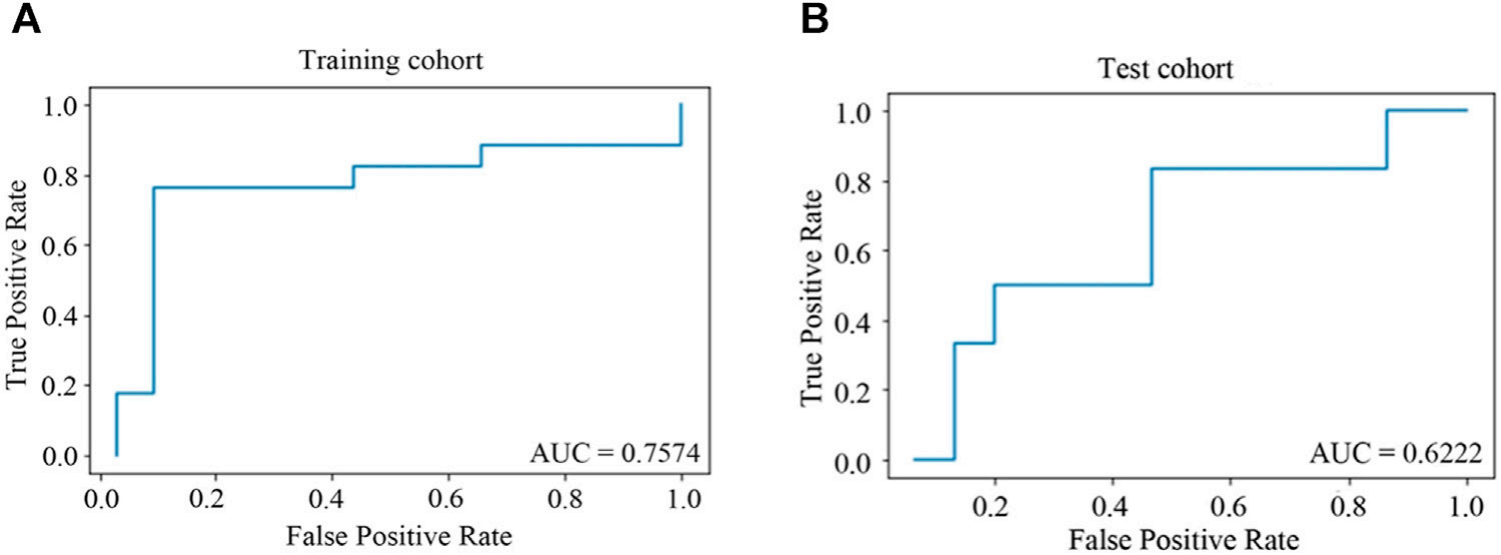
ROC curves for predictors of HD-MTX-induced liver injury in ridge regression model. **(A)** ROC curve in training cohort; **(B)** ROC curve in test cohort. When the value of AUC is closer to one, the classification effect of the model is better.

The model of predictors for liver injury achieved good discrimination and reached the AUC of 0.76 and 0.62 in the training and test cohorts, respectively, as depicted in Figure 3. Additional statistics representing model performance are illustrated in Table 5. The model had remarkable accuracy and precision in the training cohort (0.86 and 0.81, respectively), demonstrating its adequate capacity to predict risks accurately and precisely. The value of specificity in the training cohort was high (0.91), showing a good ability to reduce false positives. Of all metrics, the majority in the test cohort had relatively low values, possibly due to the small sample size. Nevertheless, the negative predictive value was good (0.80), indicating low likelihood that no disease was found in negative subjects.

**TABLE 5:** Performance of the ridge regression model.

Metric	Training cohort	Test cohort
Accuracy	0.86	0.57
Precision	0.81	0.36
Sensitivity	0.76	0.67
Specificity	0.91	0.53
Positive predictive value	0.81	0.36
Negative predictive value	0.88	0.80

### The Risk-Scoring Model

In the ridge regression model, the regression coefficient *βi* of each predictor was weighted and estimated. After that, the risk-score calculation formula for liver injury was obtained as follows:

Risk score = 1*age - 1*albumin_1 day before MTX administration +1 *IBIL_1 day before MTX administration - 25*MTRR_AA- 61*MTRR_AG - 44*SLCO1B1_11045879_CC + 140.

**FIGURE 4:**
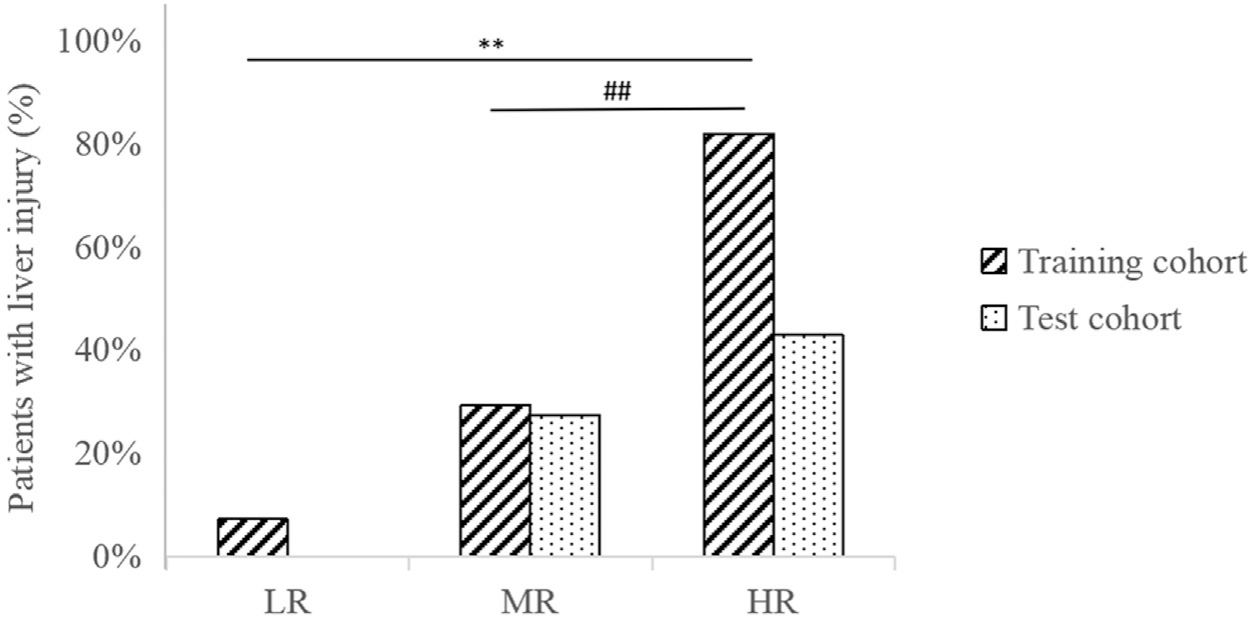
Distribution of patients with liver injury among different risk groups in training and test cohorts. **p < 0.01, indicating significant differences between LR-HR groups in the training cohort. ^##^ *p <* 0.01, indicating significant differences between MR-HR groups in the training cohort.

The data of patients in the training cohort were substituted into the formula, and the patients’ total scores were calculated, showing the score distribution with a range from 10 to 128 (Table 6). According to the upper and lower quartile levels of total risk scores, we stratified the risk scores into three groups, representing the LR (≤48), MR (48–89), and HR (>89) groups. The distribution of patients with liver injury among different risk groups in the training and test cohorts is displayed in Figure 4. We can see an ascending trend that liver injury occurred from LR to HR groups in both cohorts, and the intergroup differences of LR-HR and MR-HR in the training cohort were significant (*p* < 0.01), indicating that the model had good differentiating ability between LR-HR and MR-HR levels. However, there was no significant difference between groups in the test cohort, probably due to the limited amount of the test sample. The distribution of patients with liver injury among different risk groups was that, in the training cohort, there was one patient in the LR group, seven patients in the MR group, and nine patients in the HR group; in the test group, there were zero patients in the LR group, three patients in the MR group, and three patients in the HR group.

**TABLE 6:** Risk score description in training and test cohorts.

Risk score	Training cohort	Test cohort
Average	67.36	74.80
SD	26.38	31.61
Minimum value	10.30	0.80
Lower quartile	47.50	51.00
Median	57.90	78.20
Upper quartile	88.65	98.60
Maximum value	128.20	126.50

**Abbreviation**: SD, standard deviation.

## Discussion

The present study focuses on investigating the associations between clinical, genetic, and laboratory factors and HD-MTX-induced liver injury in pediatric ALL patients in China, constructing a risk-scoring model and endeavoring to achieve a balance between the efficacy and toxicity of HD-MTX treatment.

The identification of important factors is crucial, and multiple covariates have demonstrated their significance in previous studies. A population pharmacokinetic model was constructed for ALL children, finding age and total body weight as significant influencing factors in MTX clearance (Aumente et al., 2006). It illustrates an inverse relationship between MTX clearance and patient age, which means younger patients show faster elimination of HD-MTX, leading to maximum treatment effect without elevating toxicity (Aumente et al., 2006). Based on the predictor coefficients in our risk-scoring formula, age is positively related to the risk of hepatotoxicity caused by HD-MTX, which is consistent with former perspectives. In terms of other nongenetic factors, the elevation of ALT and AST could indicate potential damaged liver structural integrity (Seidel et al., 1994; Elbarbary et al., 2016; Hakamata et al., 2018). ALP and GGT are common markers of cholestatic problems induced by liver injury (Hawkey et al., 2012). However, these transaminases were not included in the final model; likewise, kidney function indexes, including Cr, urine protein, urine pH, and BUN, were not selected as predictors, possibly due to the initial exclusion of patients with abnormal liver and/or kidney function. In addition, serum bilirubin is related to the excretion of anions and formation of bile, and albumin has an impact on protein synthesis (Limdi and Hyde, 2003). HD-MTX is proven to increase the concentration of serum bilirubin while decreasing albumin concentration, corresponding to our findings that the HD-MTX-induced liver injury risk was positively correlated to IBIL and negatively to albumin (Moghadam et al., 2015).

Of all risk factors for MTX toxicity, the role of gene polymorphisms is indubitable, leading to various responses to MTX toxicity among ALL children. We analyzed seven genes from MTX metabolism, some of which have had their impacts on MTX-induced toxic reactions illustrated in previous reports; these include that *MTHFR_C677T* can significantly increase the risk of MTX toxicity, the low activity of *GGH* may lead to increased intracellular cytotoxicity of MTX in leukemic cells, the inhibition of *DHFR* can be definitively responsible for the exertion of MTX cytotoxicity, and *SHMT1* can affect enzymatic activity, which is associated with liver toxicity during MTX therapy (Fotoohi and Albertioni, 2008; Schmiegelow, 2009; Lopez-Lopez et al., 2011; Yang et al., 2012). In the final results, *MTRR* and *SLCO1B1* polymorphisms occupied a critical position in the risk-score formula with significantly greater weightings than other predictors, and their negative coefficients represent an inverse relationship between *MTRR* and *SLCO1B1* polymorphisms and liver injury risks. In other words, patients with *MTRR_AA, MTRR_AG*, and/or *SLCO1B1_11045879_CC* could have lower risk of liver injury. The present work provides a novel perspective about the effect of *MTRR* polymorphisms on the HD-MTX toxicity in ALL children. In terms of the *SLCO1B1* gene, it was deemed to be associated with MTX clearance, and the significant correlations between its polymorphisms (*rs11045879* and *rs4149056*) and the levels of serum MTX are proven in other studies (Faganel Kotnik et al., 2011; Niemi et al., 2011; Moriyama et al., 2015).

There are studies pointing out that some gene polymorphisms involved in the MTX pathway that are not investigated in our study may have associations with MTX toxicity as well. A polymorphism in reduced folate carrier (*RFC1*), *C3435T* in the multidrug-resistance protein (*ABCB1*), and *C421A* in the breast cancer resistance protein (*ABCG2*) are reported to have associations with hepatic, gastrointestinal, and nervous system toxicities (Lopez-Lopez et al., 2011; Mikkelsen et al., 2011). However, there is an adverse viewpoint from Lopez et al. with a research population of 115 ALL children, who claimed that *MTHFR* (*C677T* and *A1298C*), *TYMS* (*28 bp* and *6 bp-del*), *SHMT1* (*C1420T*), *ABCB1* (*C3435T*), *ABCG2* (*C421A*), *RFC1* (*G80A*), and *SLCO1B1* (*rs4149081*) were not available to show relationships with MTX toxicity in childhood ALL (Lopez-Lopez et al., 2011). They believe that the associations between polymorphisms in enzymes or transporter genes and MTX proven in the former literature could be attributed to the small or unhomogeneous sample size, nonobjective toxicity markers, or different treatment protocols (Lopez-Lopez et al., 2011). Herein, we analyzed as many as 10 polymorphisms in seven genes, which provides a comprehensive analysis of the associations between genotypes and HD-MTX-induced liver injury, to provide an optimized therapy regimen based on individual diversity. Moreover, we chose quantifiable markers of toxicity, ALT, and AST levels to objectively analyze HD-MTX-induced hepatotoxicity. However, we were aware that one drawback in this study was the inadequate sample size; thus further research with larger populations and clinical practice are necessary.

In summary, we explored the relationship between multiple risk factors and HD-MTX-induced liver injury and established a risk-scoring model based on the identified predictors. Besides liver injury, the modeling process in present study can be applied to predict the risks of other MTX-induced ADEs caused by MTX. This study provides knowledge of risk prediction for HD-MTX hepatotoxicity in pediatric ALL patients that few studies have investigated, which could be used as a reference for clinical medication.

## Data Availability

The raw data supporting the conclusions of this article will be made available by the authors, without undue reservation.
