# Supplementary material for: A Risk Scoring Model for High-Dose Methotrexate-Induced Liver Injury in Children With Acute Lymphoblastic Leukemia Based on Gene Polymorphism Study
Source: Front Pharmacol. 2021 Sep 29;12:726229. doi: 10.3389/fphar.2021.726229 (PMC8511303; doi:10.3389/fphar.2021.726229)
Supplement: Supplementary file 1 [file Table1.DOCX]

Supplementary Tables:

**Table S1. Detailed information on genotyping analysis.**

| **Origin** | **Gene** | **Serial number** | **Genotype** | **Probe/primer** | **5’-3’ sequence** |
| --- | --- | --- | --- | --- | --- |
| China | MTHFR | rs1801133 | CC | F | ATTGGCAGGTTACCCCAAAG |
|  | C677T |  | CT | R | ATGCCTTCACAAAGCGGAAG |
|  |  |  | TT | A | VIC-GTGTCTGCGGGAGTCG-NFQ-MGB |
|  |  |  |  | G | FAM-GTGTCTGCGGGAGCCG-NFQ-MGB |
| China | MTHFR | rs1801131 | AA | F | CTGAAGAGCAAGTCCCCCAAG |
|  | A1298C |  | AC | R | CACTCCAGCATCACTCACTTT |
|  |  |  | CC | A | VIC-AGCTGACCAGTGAAGCA-NFQ-MGB |
|  |  |  |  | G | FAM-AGCTGACCAGTGAAGAA-NFQ-MGB |
| China | SLCO1B1 | rs2306283 | AA | F | TGTTTAATTCAGTGATGTTCTTACAGTTACA |
|  | *1b |  | AG | R | AATATTAATTCTTACCTTTTCCCACTATC |
|  |  |  | GG | A | VIC-ATTCTAAAGAAACTAATATCGATT-NFQ-MGB |
|  |  |  |  | G | FAM-ATTCTAAAGAAACTAATATCAATT-NFQ-MGB |
| China | SLCO1B1 | rs4149056 | TT | F | CTACATAGGTTGTTTAAAG |
|  | *5 |  | CT | R | AGCGAAATCATCAATGTAAGAAAG |
|  |  |  | CC | A | VIC-ACATGTGGATATATGCGT-NFQ-MGB |
|  |  |  |  | G | FAM-CATGTGGATATATGTGT-NFQ-MGB |
| China | MTRR | rs1801394 | AA | F | CCCATTTTTCAGTTTCACTGTTA |
|  |  |  | AG | R | TCAAAGCACAAAACGGTAAAATCCACT |
|  |  |  | GG | A | VIC-TCGCAGAAGAAATGTG-NFQ-MGB |
|  |  |  |  | G | FAM-TCGCAGAAGAAATATG-NFQ-MGB |
