# Supplementary material for: A Risk Scoring Model for High-Dose Methotrexate-Induced Liver Injury in Children With Acute Lymphoblastic Leukemia Based on Gene Polymorphism Study
Source: Front Pharmacol. 2021 Sep 29;12:726229. doi: 10.3389/fphar.2021.726229 (PMC8511303; doi:10.3389/fphar.2021.726229)
Supplement: Supplementary file 2 [file Table2.DOCX]

Supplementary Tables:

**Table S2. MTX plasma concentration measured at 48h and 72h.**

| **Patient No.** | **MTX plasma concentration** | |
| --- | --- | --- |
|  | **C_48h_** | **C_72h_** |
| 1 | 0.29 | <0.1 |
| 2 | 0.68 | 0.1 |
| 3 | 0.35 | 0.16 |
| 4 | 0.78 | <0.1 |
| 5 | 0.12 | <0.1 |
| 6 | 0.37 | 0.12 |
| 7 | 11.2 | 3.75 |
| 8 | 0.38 | 0.19 |
| 9 | 0.55 | <0.1 |
| 10 | 4.5 | 1.63 |
| 11 | 0.45 | 0.13 |
| 12 | Missing | <0.1 |
| 13 | 0.28 | <0.1 |
| 14 | 0.1 | <0.1 |
| 15 | 0.59 | <0.1 |
| 16 | 0.39 | <0.1 |
| 17 | 0.57 | 0.21 |
| 18 | 1.36 | 0.8 |
| 19 | 0.49 | 0.2 |
| 20 | 0.18 | <0.1 |
| 21 | 0.3 | 0.13 |
| 22 | 0.5 | <0.1 |
| 23 | 0.55 | <0.1 |
| 24 | 0.1 | <0.1 |
| 25 | 0.42 | <0.1 |
| 26 | 0.44 | 0.13 |
| 27 | 0.15 | <0.10 |
| 28 | 0.24 | <0.10 |
| 29 | 0.39 | 0.12 |
| 30 | 0.64 | 0.22 |
| 31 | 0.16 | <0.10 |
| 32 | 0.32 | 0.16 |
| 33 | 1.14 | 0.25 |
| 34 | 0.11 | 0.1 |
| 35 | 0.12 | 0.1 |
| 36 | 0.78 | 0.29 |
| 37 | 0.36 | 0.2 |
| 38 | 0.24 | 0.13 |
| 39 | 0.64 | 0.38 |
| 40 | 0.32 | 0.16 |
| 41 | 0.28 | 0.1 |
| 42 | 0.24 | 0.23 |
| 43 | 15 | 0.52 |
| 44 | 0.32 | 0.27 |
| 45 | 0.62 | 0.19 |
| 46 | 0.36 | <0.1 |
| 47 | 0.57 | <0.1 |
| 48 | 0.39 | 0.16 |
| 49 | 0.3 | <0.1 |
| 50 | 0.33 | 0.11 |
| 51 | 0.2 | <0.1 |
| 52 | 1.53 | <0.1 |
| 53 | 0.49 | <0.1 |
| 54 | 0.67 | 0.22 |
| 55 | 0.37 | <0.1 |
| 56 | 4.2 | 1.42 |
| 57 | 0.22 | <0.1 |
| 58 | 0.94 | 0.16 |
| 59 | 1.19 | 0.33 |
| 60 | 0.42 | <0.1 |
| 61 | 2.35 | 0.8 |
| 62 | 0.25 | <0.1 |
| 63 | 0.43 | <0.1 |
| 64 | 0.95 | 0.16 |
| 65 | 2.1 | 0.88 |
| 66 | 1.31 | <0.1 |
| 67 | 0.25 | <0.1 |
| 68 | 0.24 | <0.1 |
| 69 | 0.23 | <0.1 |
| 70 | 0.14 | <0.1 |
